# Research Progress on the Microenvironment and Immunotherapy of Advanced Non-Small Cell Lung Cancer With Liver Metastases

**DOI:** 10.3389/fonc.2022.893716

**Published:** 2022-07-28

**Authors:** Yujia Fang, Chunxia Su

**Affiliations:** Department of Oncology, Shanghai Pulmonary Hospital, Shanghai, China

**Keywords:** NSCLC, liver metastases, lung cancer, immunotherapy, microenvironment

## Abstract

Lung cancer is a malignant tumor with the highest morbidity and mortality, and more than 75% of patients are diagnosed at an advanced stage. Liver metastases occur in 20% of non-small cell lung cancer patients, and their prognosis are poor. In recent years, immune checkpoint inhibitor monotherapy and combination therapy have made breakthrough progress in advanced Non-small cell lung cancer (NSCLC) patients. However, compared with the overall population, the liver metastases population was an independent prognostic factor for poor immunotherapy response. Whether and how immunotherapy can work in NSCLC patients with liver metastases is a major and unresolved challenge. Although more and more data have been disclosed, the research progress of NSCLC liver metastasis is still limited. How liver metastasis modulates systemic antitumor immunity and the drug resistance mechanisms of the liver immune microenvironment have not been elucidated. We systematically focused on non-small cell lung cancer patients with liver metastases, reviewed and summarized their pathophysiological mechanisms, immune microenvironment characteristics, and optimization of immunotherapy strategies.

## Introduction

Lung cancer is the most common malignancy and the most leading cause of cancer-related mortality worldwide. Non-small cell lung cancer (NSCLC) accounts for about 80%~85% of the total lung cancer population. Metastasis is a primary driver of NSCLC-related mortality, with the liver representing frequently involved organ. According to epidemiological studies, the incidence of NSCLC liver metastases is about 4%. The prognosis of patients with liver metastases from lung cancer is poor with a median overall survival of only about 4 months (1). It would be worthwhile to explore better treatment options for these population. Checkpoint inhibitor immunotherapies bring transformative advances in cancer treatment, benefiting numerous patients. However, more and more clinical research data prove that the liver metastases population has a poor response to immunotherapy. The KEYNOTE-001 clinical study suggests that patients with liver metastases can also benefit from immune checkpoint inhibitor (ICI) therapy, but liver metastases are still an independent predictor of poor immunotherapy efficacy compared with the overall population [liver metastases vs no liver metastases progression free survival (PFS): 1.8 months vs 4.0 months, P<0.05] (2).Whether and how immunotherapy work in NSCLC patients with liver metastases is a major and unresolved challenge. How liver metastasis modulates systemic antitumor immunity and the drug resistance mechanisms of the liver immune microenvironment have not been elucidated.

We systematically focused on non-small cell lung cancer patients with liver metastases, reviewed and summarized their pathophysiological mechanisms, immune microenvironment characteristics, and optimization of immunotherapy strategies.

## Pathophysiological Mechanisms of Liver Metastases

The liver is composed of 80% hepatocytes and 20% non-parenchymal cells. It has unique anatomical properties. In the liver, oxygen-rich blood from the hepatic artery mixes with venous blood from the portal vein in the sinusoid. Liver metastases in different tumors are influenced by multiple factors, such as blood flow patterns, tumor stage, and tumor histological subtype. The occurrence of liver metastases from lung cancer is usually thought to be caused by tumor cells through the portal venous circulation to seed the liver primarily. There are of course other factors, such as the seed and soil hypothesis related to histocompatibility and homing mechanisms (3). It is generally believed that liver metastases are mainly divided into four stages as circulating tumor cells enter the hepatic sinusoids, including a microvascular phase; an extravascular/pre-angiogenic phase; an angiogenic phase; a Growth phase.

### Microvascular Phase

At this stage, circulating tumor cells pass through the portal vein into the sinusoidal. First confronted with liver-specific defense mechanisms, tumor cells encounter Kupffer cells (KCs), natural killer cells (NKs), and liver sinusoidal endothelial cells. Immune cells will kill it and cause it to die.

Kupffer cells can cause transient hepatic sinusoidal blood flow blockage by phagocytosing cancer cells, and the inflammatory response triggered by ischemia-reperfusion injury can further activate KC and release a variety of cytokines [such as interleukin-1 (IL-1), interleukin-6 (IL-6)] and chemokines (such as macrophage-inflammatory protein-2 (MIP-2), monocyte chemoattractant monocyte chemotactic protein-1 (MCP-1)],thereby activating non-specific inflammatory cells such as NK cells and neutrophils, and enhancing local anti-tumor effects.

It should be noted that the local inflammatory response can induce liver sinusoidal endothelial cells (LSEC) to express key cell adhesion proteins E-selectin, P-selectin, etc., which mediate the direct adhesion of cancer cells through the interaction with receptors. such as death receptor 3.

In addition, tumor cell-platelet interactions can also lead to disruption of endothelial cell junctions, which in turn promotes their metastasis. Inhibition of platelet function may inhibit metastasis. Relevant preclinical studies have shown that aspirin reduces metastasis by inhibiting platelet COX-1 and its product TXA, but there is a certain risk of clinical complications (4).

### Extravascular/Pre-Angiogenic Phase

As tumor cells extravasate from the sinusoidal microvasculature into the Disse space, hepatic stellate cells (HSCs) are activated by a pro-inflammatory cascade that begins at the microvascular stage (5).At the same time, HSCs produce extracellular matrix (ECM) proteins, including fibronectin, collagen, laminin, and more. HSCs produce growth factors such as TGFβ and pro-angiogenic factors such as VEGF and angiopoietin 1, and release chemokines and cytokines to recruit inflammatory or immune cells (6).KCs and neutrophils secrete matrix metalloproteinases (MMPs) and elastases, and ECM proteins and ECM-degrading proteases, including MMPs, stimulate angiogenesis by providing a scaffold for migrating endothelial and cancer cells.

### Angiogenic Phase

Hypoxia inhibits tumor growth and thus requires angiogenesis to overcome the hypoxic immune microenvironment of tumors. Interactions between established niches and tumor cells are important for the induction of angiogenesis. Hypoxic cells secrete angiogenic factors, including vascular endothelial growth factor (VEGF) A. VEGF-A mediates endothelial cell migration and promotes the formation of new blood vessels. ICAM-1 expressed on LSECs promotes the secretion of IL-6, prostaglandin E2, VEGF and MMP2 by tumor cells, which in turn induces HSCs to secrete VEGFA and MMP2. These cytokines stimulated the migratory and angiogenic potential of LSECs and HSCs. A variety of cell types can aid in metastatic colonization by secreting pro-angiogenic factors.

### Growth Phase

Growth of vascularized micro-metastases leads to tumor cell proliferation and establishment of clinically detectable metastases. With the release of VEGF factors, recruit innate agents that may have tumor suppressor (M1 macrophages, N1 neutrophils, NK cells) or tumor promoting (M2 macrophages, myeloid-derived suppressor cells (MDSC)) effects and adaptive immune cells, activate dendritic cells, induce T cells to interact with tumor expansion. With the formation of new blood vessels and the improvement of the hypoxic environment of the tumor, the tumor gradually develops into large metastases (7).

## Liver Microenvironment

### Myeloid-Derived Suppressor Cell

MDSCs originate from the immature myelocytic suppressor population of the bone marrow. Many tumor-related factors can induce the infiltration of MDSCs in the hepatic immune microenvironment, such as tumor-derived granulocyte colony-stimulating factor (G-CSF) and granulocyte/macrophage colony-stimulating factor (GM-CSF). Induction of MDSC infiltration in tumor tissue; the local hypoxic state caused during tumor cell proliferation can also promote the accumulation of MDSC in the microenvironment. MDSC can induce the enrichment and proliferation of Treg, inhibit the function of dendritic cells and NK cells, and promote the polarization of the macrophage population from M1 to M2 (7).In the liver immune microenvironment, MDSCs express galectin-9. It acts as a ligand for T-cell immunoglobulin and mucin domain 3 and induces apoptosis. Preclinical studies in hepatocellular carcinoma have shown that MDSCs may be a potential therapeutic target for hepatic immune tolerance, and the combination of MDSC-targeted inhibitors and ICI can prolong the survival rate of hepatocellular carcinoma mice (8, 9).In a recently published data showing that MDSC infiltration and CD8+ T cell infiltration are independent predictors of recurrence-free survival and overall survival, respectively, and high MDSC infiltration is associated with poor clinical outcomes in patients with hepatocellular carcinoma, further suggesting that MDSCs play a role in The liver microenvironment may play an inhibitory role, and targeting this immune cell target may be a promising development direction for immunotherapy drugs (10, 11).

### Tumor-Associated Macrophages

TAMs originate from monocytes in the blood, and they are recruited to tumors by cytokines secreted by tumor cells or interstitial cells. Chemokine CCL2, CCL5, and CCL7 are all important factors involved in recruitment (12). The development of new drugs for chemokines is also a promising direction for anti-tumor therapy. TAM can be divided into M1 type activated by IFN-α/β or IFN-γ and M2 type activated by IL-4/IL-10 according to different activation pathways. M1-type TAMs produce anti-tumor factors TNF-α and NO, stimulate T cells to induce activation and play an anti-tumor immune response. M2-type TAMs can produce tumor-promoting factors such as IL-6 and VEGF, MMP-2, MMP-7 to promote angiogenesis, thereby promoting tumorigenesis (13, 14).The ratio of M1/M2 TAMs varies among individuals and in different immune microenvironments. In clinical studies of hepatocellular carcinoma, it was found that low presence of CD86+ TAM and high presence of CD206+ TAM in hepatocellular carcinoma tumor tissue were associated with advanced clinical stage, poor overall survival (OS) and increased time to recurrence (TTR) significantly correlated, implying a worse prognosis (1).While cell-derived Wnt ligands stimulate M2-like cells to induce TAM polarization through the Wnt/β-catenin pathway, blocking Wnt signaling in cancer cells or Wnt/β-catenin pathway activation in TAM may be a feasible research direction in the future (14–17).

### Regulatory T cells

Tregs play an important role in the immune microenvironment of the liver. It has been a hot research topic in recent years about how it functions in the microenvironment, how it infiltrates and metastasizes to tumor sites, and how it mediates immune tolerance.Foxp3+ Tregs can suppress deleterious immune responses and, under normal physiological conditions, prevent autoimmunity by downregulating IL-2, releasing adenosine, and secreting immunosuppressive cytokines including TGF-β, IL-10, and IL-35,which regulate the immune response, thereby inhibiting the occurrence of autoimmune diseases (18, 19).On the other hand, Tregs suppress protective immune responses against invading pathogens or tumors, leading to further disease progression. In previous studies, Tregs characterized by CD25 and FoxP3 expression were found to be potent mediators of immunosuppression in the tumor immune microenvironment, and their presence in the TME was associated with increased metastasis and poor outcome in many malignancies (20, 21).Interaction of T cell receptors (TCRs) with IL-10 and TGF-β signaling promotes the infiltration of Tregs into the tumor microenvironment by modulating the CCL6/CCL20 axis (22). In hepatocellular carcinoma, tumor cells secrete CCL5, CCL22 and CCL28 chemokines to mediate Treg accumulation (19). At the same time, in clinical studies of patients with hepatocellular carcinoma, it was found that the number of Treg in the immune microenvironment of patients was much higher than that of healthy controls, and the up-regulation of Treg expression was correlated with the shortening of progression-free survival of patients. Notably, Tregs express surface molecules such as CTLA-4 and PD-1, which means that they may be direct targets for ICI immunotherapy. However, reports of clinical trials of immunotherapy targeting Tregs are rare (23).

### Tumor-Associated Neutrophil

As the most abundant circulating white blood cells in human blood, neutrophils are considered to be the first line of defense in innate immune defense (24). TAN is generally believed to be differentiated from MDSCs and is considered to be one of the emerging targets of various cancer types in the tumor immune microenvironment. Inhibiting the recruitment and activity of neutrophils may improve the tumor microenvironment and play a therapeutic role (25). In the immune microenvironment of NSCLC, myeloid cells make up 50% of tumor-infiltrating CD45+ cells and TANs make up 20% of CD45+ cells. A retrospective analysis of clinical data found that in patients with advanced metastatic disease, the pretreatment neutrophil-to-lymphocyte ratio was associated with shorter OS and PFS, predicting a worse prognosis (26). This also suggests that TAN may be a potential prognostic biomarker. TAN can generally be divided into two cell subtypes, N1 and N2. N1 TANs can increase tumor cytotoxicity, increase the secretion of immune-activating cytokines, and mainly exert anti-tumor effects. N2 TANs can promote angiogenesis and tumor cell proliferation, and secrete tumor necrosis factor TNFα, epidermal growth factor to play a tumor-promoting role (27). In the early stages of tumor development, N1 TANs predominate, while N2 TANs accumulate gradually as the tumor progresses. The differentiation of different subtypes of TAN may be affected by transforming growth factor β, but the specific mechanism has not been clearly explored. In the TME, TAN can promote or inhibit tumor development by regulating cell growth factors. In hepatocellular carcinoma, tumor cells and TAN work together to activate neutrophils in peripheral blood of patients, secrete chemokine CC motif ligand 2 (CCL2) and chemokine CC motif ligand 17 (CCL17), and then promote tumor growth, proliferation, and macrophage growth. The infiltration of phagocytes and Treg in tumors ultimately promotes tumor growth. The differentiation of TAN can be regulated by regulating cytokines and chemokines in the immune microenvironment, so that it mainly plays an anti-tumor effect in the liver immune microenvironment (28–30).

### Liver Sinusoidal Endothelial Cells

Liver sinusoidal endothelial cells (LSEC) are unique cells in the liver microenvironment. It expresses major histocompatibility complex I (MHC I) and II (MHC II) proteins, co-stimulatory molecules CD40, CD80, CD86, and plays a major role in the clearance and antigen presentation in the immune microenvironment of the liver (31). Its main function is to remove blood-borne wastes from the systemic circulation and digestive tract through filtration and endocytosis (32). Under some pathological conditions, LSECs undergo capillarization by losing their fenestration and developing a basement membrane (33). At the same time, capillarized LSECs will release PDGF and decreased expression of vasoprotective Kruppel-like factor 2 to active of liver stem cells which can give rise to tumor cells after malignant transformation (34). LSEC presents exogenous antigens to CD8+ T cells in the form of MHC I molecules, and the functions of CD8+ T cells activated by LSEC are mostly inhibited. Preclinical studies in mice have found that CD8+ T cells activated by LSEC do not secrete interleukin-2 (IL-2), and exogenous supplementation of IL-2 can relieve the immune tolerance of T cells., activating the immune microenvironment, which may be one of the mechanisms responsible for the special immune microenvironment of the liver (35).

### Cytokines

The immune microenvironment of liver metastases from non-small cell lung cancer is also affected by the joint action of multiple cytokines.

IL-6 is a pleiotropic cytokine that can bind to cell surface-expressed IL-6 receptors or form complexes with soluble IL-6 receptors, which signal to gp130, termed “IL-6 trans- Signaling”. IL-6 is up-regulated in a variety of tumors, and exerts both pro- and anti-inflammatory functions in the immune microenvironment, depending on the cell type and at different stages (36, 37).Inhibition of IL-6 and STAT3 signaling pathways accelerates tumor development in mice with hepatocellular carcinoma in preclinical studies in hepatocellular carcinoma. Depleting IL-6 and STAT3 signaling resulted in an accumulation of hepatic steatosis, macrophage recruitment, and increase of hepatocyte proliferation (38).However, there are also different preclinical findings showing that macrophages release IL-6 receptors to induce IL-6 trans-signaling, IL-6 trans-signaling inhibits p53-mediated cell death and activates β-catenin Signaling pathway promotes hepatocellular carcinoma progression in mice (39). Targeted deletion of IL-6 in macrophages reduced the incidence of spontaneous liver cancer by disrupting the IL-6/STAT3 axis, promoting cell proliferation and cell death resistance (40).

TGF-β plays different roles at different stages of the liver tumor immune microenvironment. In the process of tumor occurrence and development, it often plays an anti-tumor effect at the initial stage, but plays a tumor-promoting function after the tumor colonization progresses (41).Results of a preclinical study showed that blocking TGFβ signaling sensitized tumors to anti-PD-1-PD-L1 therapy in colon cancer mice with liver metastatic disease. Increased TGFβ in the tumor microenvironment promotes T cell rejection and prevents the development of the TH1 effector phenotype, further creating an immune tolerance microenvironment. Inhibition of TGFβ unleashes a potent and durable cytotoxic T-cell response against tumor cells, preventing metastasis (42). Overexpression of Smad3, a downstream target of TGF-β, inhibits tumor growth by downregulating the anti-apoptotic protein Bcl-2. TGF-β also plays an inhibitory role in inducing tumor cell senescence by promoting the accumulation of Nox4 and reactive oxygen species (ROS). SMAD4 is also an essential molecule in the TGF-β signaling pathway. It has been reported that up to 62% of colorectal cancer patients with liver metastases have SMDA4 downregulation (43–45).

Overall, the tumor immune microenvironment of non-small cell lung cancer liver metastases is mediated by the interaction of different types of cells and intricate cytokines and related pathways. As the tumor continues to grow, various components of the immune microenvironment will interact with tumor cells to disrupt the balance: for example, CAF, LSECs and myeloid cells secrete growth factors to promote tumor growth and migration; TAM and TAN interact with each other. The effect leads to the depletion of T and NK cells, which together lead to the formation of an immune tolerance microenvironment in the liver. By understanding the roles played by various cells and factors in the tumor immune microenvironment, it will help to better understand the formation of the immune tolerance microenvironment, and help to find suitable therapeutic targets or pathways to improve the immune microenvironment of liver metastases.

## Immunotherapy Strategy Optimization

Tumeh et al. reported a result in 2017 that in a population of melanoma and NSCLC patients treated with the anti-PD-1 antibody pembrolizumab, immunotherapy in patients with liver metastases was less effective than those without liver metastases [PFS: 5.1 months vs 20.1months, objective response rate (ORR): 30.6% vs 56.3%, P<0.0001] (46) Liver metastases are associated with reduced CD8+ T cell density at the margins of infiltrating tumors(liver metastases group mean count 547, non-liver metastases group, mean count 1,441; P < 0.016), which may account for the poor response to immunotherapy. Analysis of the Phase 1 CA209-003 trial in 2019, reported in a population that included patients with melanoma, renal cell carcinoma, and non-small cell lung cancer, the presence of liver metastases was independently associated with reduced likelihood of survival at 5 years[odds ratio (OR)= 0.31; 95% CI, 0.12-0.83; P =0 .02] (47) But it is worth noting that KEYNOTE001 clinical study suggests that patients with liver metastases may also benefit from ICI therapy. However, liver metastases remained an independent predictor of poor immunotherapy response compared with the overall population. Patients with liver metastases have shorter progression-free survival compared to those without liver metastases (1.8 months vs 4.0 months, P<0.05.) These evidences demonstrate that the liver metastases population is less responsive to immunotherapy relative to the general population (2).

Considering the poor efficacy of immune monotherapy for liver metastases which has also been validated in other solid tumors, it may be worth looking forward to actively exploring immune combination therapy.

## Immunotherapy Combined With Chemotherapy

The KEYNOTE-189 clinical study compared chemotherapy with pembrolizumab or placebo in patients with metastatic NSCLC. A subgroup analysis of 115 patients with liver metastases found that pembrolizumab combined with chemotherapy significantly prolonged the median OS of patients compared with chemotherapy combined with placebo (12.6 months vs 6.6 months, P<0.001). However, the HR for OS in the two groups with liver metastases (HR=0.62, 95%CI: 0.39-0.98) was similar to that in the group without liver metastases (HR=0.58, 95%CI: 0.45-0.74) (48).

One meta-analysis included 4 studies comparing immune checkpoint inhibitors combined with platinum-based doublet chemotherapy in NSCLC (49).The results showed that a significant PFS prolongation was observed in a subgroup of non-squamous NSCLC patients with liver metastases [HR = 0.63 (0.44–0.89)].But considering heterogeneity between studies as high as I2 = 57%. Further analysis found that two studies of atezolizumab in combination with chemotherapy showed a more significant response in this population [HR = 0.85 (0.61–1.19)]. It was inferred that in NSCLC patients with liver metastases, atezolizumab may prolong PFS. Another Meta-analysis evaluated the effect of PD-1/PD-L1 inhibitor combined with chemotherapy on the first-line treatment effect of lung cancer patients with liver metastases which included 8 randomized controlled clinical studies. The results showed that PD-1/PD-L1 inhibitor combined with chemotherapy can reduce the risk of tumor progression (HR=0.60, 95% CI: 0.55-0.65) and the risk of death (HR=0.71, 95% CI: 0.58-0.90) (50).

## Combination of Immunotherapy and Anti-Tumor Angiogenesis

A retrospective study reported in World Conference on Lung Cancer(WCLC) in 2021 evaluating the outcomes of the IMpower150 regimen (atezolizumab + bevacizumab + carboplatin + paclitaxel) in patients with stage IV non-squamous NSCLC (Abstract No: P16. 02), of the 54 patients with stage IV NSCLC included, 23 (43%) patients had liver metastases at baseline. The ORRs for the liver metastases population and the overall population were 61% and 58%, respectively. The overall population PFS was 5.1 months and overall survival was 8.3 months.

Meanwhile, in the IMpow150 study, the first-line treatment of metastatic NSCLC patients without chemotherapy, it was found that ABCP group(atezolizumab + bevacizumab + carboplatin + paclitaxel)was significantly better than BCP group(bevacizumab + carboplatin+paclitaxel)significantly prolonged the OS of patients (19.5 months vs 14.7 months, P=0.01, HR=0.80, 95% CI: 0.73-0.95). The analysis of liver metastasis subgroup showed that the OS of the ABCP group was 4.1 months longer than that of the BCP group (13.2 months vs 9.1 months, P<0.01, HR=0.67, 95% CI: 0.45-1.02). In general, the four-drug combination regimen of ABCP can benefit for patients with advanced NSCLC. For the subgroup of liver metastases, considering the limited sample size, further studies with larger sample sizes are expected in the future (51).

A retrospective study investigated the efficacy and safety of anti-PD-1 combined with anlotinib in patients with advanced non-small cell lung cancer after failure of prior systemic therapy. For the overall patients, the median PFS was 6.9 months (95% CI 5.5–8.3 months), and median OS was 14.5 months (95% CI, 10.9–18.1 months). The patients with liver metastasis had a mPFS of 6.9 months and a mOS of 11.9 months (51). It is worth noting that this study had no control group and there were differences in the use of immune checkpoint inhibitors, so the results need to be further corroborated by a larger randomized controlled cohort study.

## Immunotherapy Combined With Radiotherapy

Preclinical studies have shown that in subcutaneous tumor models, radiation therapy and immunotherapy can synergistically contribute to the improvement of ICI efficacy (52, 53).

A study in Nature Medicine reports that liver-directed radiation therapy can reshape the liver immune microenvironment and restore the effects of immunotherapy in models of liver metastases. In a retrospective study of clinical cohorts, the researchers found that the presence of liver metastases at baseline was associated with a diminished response to immunotherapy, but not targeted therapy and chemotherapy. Mouse models further demonstrate that liver metastasis recruits and polarizes monocyte-derived macrophages, promotes systemic loss of antigen-specific T cells, and modulates immune function by altering the hepatic immune microenvironment. In contrast, liver-directed radiotherapy increased hepatic T-cell infiltration, decreased hepatic myeloid numbers, and decreased the ratio of CD11b+F4/80+ myeloid/CD8+ T cells; in addition, combination therapy Enhanced therapeutic effect on Ki67+, gamma-interferon and granzyme B+ cd8 T cells. These studies demonstrate that liver-directed radiation therapy can simultaneously block the myeloid components of immunosuppression, stimulate liver T-cell immunity, reshape the immune microenvironment, and restore the antitumor effect of immunotherapy (54)

Tumor irradiation can increase antigen expression and exposure, and has been found to increase T cell repertoires in preclinical models and patients (55). Radiation therapy can act as an immune adjuvant by inducing the formation of tumor micronuclei, generating cytoplasmic DNA, and causing lipid oxidation that stimulates immune responses (56). Preclinical studies have shown that mice receiving local radiotherapy in the liver have a higher proportion of T cells inducible T-cell costimulatory (ICOS), glucocorticoid-induced tumor necrosis factor receptor (GITR) and LAG3 CD8+ T cells and CD4+ T cells expressing 4-1BB, GITR and TIM-3 also expressed higher levels of PD-1/PD-L1 on the tumor cell surface, to promote systemic anti-tumor immunity (57–59).

A clinical study in solid tumors, including NSCLC, investigating the efficacy and safety of the anti-CTLA-4 antibody ipilimumab and SBRT. Results of the study found that SBRT targeting a single disease site in the lung or liver and administered concurrently or sequentially with ipilimumab resulted in partial responses or stable disease lasting ≥6 months in 23% of patients (60).

The PACIFIC study (61)aimed at patients with stage III NSCLC and explored the efficacy of receiving ICI as a consolidation regimen after concurrent chemoradiotherapy. The results showed that durvalumab could significantly improve the median PFS of patients compared with placebo (16.8 months vs 5.6 months, HR=0.68, 95% CI:0.469~0.997, P=0.00251).

The combination of radiotherapy and immunotherapy has certain clinical prospects for non-small cell lung cancer. However, the optimal timing and sequence of using this combination requires further exploration. At present, relevant clinical studies are in progress, and the follow-up data are expected to be released.

## Summary and Outlook

Non-small cell lung cancer patients with liver metastases, as a special population of advanced lung cancer, have the characteristics of poor immunotherapy. At the same time, there are limited clinical studies on this special population, and it is difficult to explore suitable immunotherapy methods through subgroup analysis in large clinical studies. By reviewing preclinical research, summarizing the pathophysiological mechanisms of non-small cell lung cancer liver metastases, and in-depth study of the mechanisms of different immune cells in the liver immune microenvironment are of great significance for the development of new therapeutic strategies for patients with liver metastases. The tumor microenvironment has important implications in determining the fate of antitumor immune responses. The special and complex immune tolerance microenvironment of the liver is closely related to the diverse immune cell populations in the microenvironment. Cell populations may play different roles in different stages of tumorigenesis and development. At the same time, cells are also affected and interacted by cytokines, chemokines, etc. At present, the research on the immune microenvironment of the liver has relatively in-depth research in the population of hepatocellular carcinoma and colorectal cancer liver metastases, and the microenvironment research on the specific population of lung cancer liver metastases is very limited. We tried to summarize the possible roles and mechanisms of immune cells in the microenvironment reported in preclinical studies, which is conducive to further exploration of lung cancer patients with liver metastases.

At the same time, immunotherapy has brought hope of long-term survival to patients with advanced lung cancer. It is of great significance to seek a possible combination therapy in view of the clinical status of the poor effect of immune monotherapy in patients with liver metastases from non-small cell lung cancer. We summarized the clinical related research progress of immune combined chemotherapy, immune antivascular therapy, and immune radiotherapy. In general, atezolizumab combined with bevacizumab can significantly improve the survival benefit of patients with liver metastases from NSCLC, and it is expected to become a new standard first-line treatment treatment solutions. Immune combined radiotherapy may also be a more suitable regimen for patients with liver metastases. In general, in the future, the synergistic and antagonistic effects of different treatment regimens should be considered from the mechanism, the optimal timing and immunotherapy regimens should be explored, and immunotherapy strategies should be optimized to seek to better improve the prognosis of this population, so that this population Can also benefit from immunotherapy.

In conclusion, more efforts should be made to understand the biological information of the immune microenvironment in the NSCLC population with liver metastases, and more clinical studies should be conducted to help develop better treatment strategies and maximize the effectiveness of immunotherapy in this population. curative effect.

## Author Contributions

FY: Writing—Original draft, writing—review & editing. SC: Review & editing. All authors contributed to the article and approved the submitted version.

## Funding

This work was supported by National Natural Science Foundation of China (grant number: 82072568) and Shanghai Shenkang Hospital Development Center (SHDC12020110).

## Conflict of Interest

The authors declare that the research was conducted in the absence of any commercial or financial relationships that could be construed as a potential conflict of interest.

## Publisher’s Note

All claims expressed in this article are solely those of the authors and do not necessarily represent those of their affiliated organizations, or those of the publisher, the editors and the reviewers. Any product that may be evaluated in this article, or claim that may be made by its manufacturer, is not guaranteed or endorsed by the publisher.
